# Assessing intracortical myelin in the living human brain using myelinated cortical thickness

**DOI:** 10.3389/fnins.2015.00396

**Published:** 2015-10-23

**Authors:** Christopher D. Rowley, Pierre-Louis Bazin, Christine L. Tardif, Manpreet Sehmbi, Eyesha Hashim, Nadejda Zaharieva, Luciano Minuzzi, Benicio N. Frey, Nicholas A. Bock

**Affiliations:** ^1^Department of Psychology, Neuroscience and Behaviour, McMaster UniversityHamilton, ON, Canada; ^2^MiNDS Neuroscience Graduate Program, McMaster UniversityHamilton, ON, Canada; ^3^Department of Neurology, Max Planck Institute for Human Cognitive and Brain SciencesLeipzig, Germany; ^4^Department of Psychiatry and Behavioural Neurosciences, McMaster UniversityHamilton, ON, Canada

**Keywords:** cerebral cortex, myelin, magnetic resonance imaging, bipolar disorder, cortical thickness

## Abstract

Alterations in the myelination of the cerebral cortex may underlie abnormal cortical function in a variety of brain diseases. Here, we describe a technique for investigating changes in intracortical myelin in clinical populations on the basis of cortical thickness measurements with magnetic resonance imaging (MRI) at 3 Tesla. For this, we separately compute the thickness of the shallower, lightly myelinated portion of the cortex and its deeper, heavily myelinated portion (referred to herein as unmyelinated and myelinated cortex, respectively). Our expectation is that the thickness of the myelinated cortex will be a specific biomarker for disruptions in myeloarchitecture. We show representative atlases of total cortical thickness, *T*, unmyelinated cortical thickness, *G*, and myelinated cortical thickness, *M*, for a healthy group of 20 female subjects. We further demonstrate myelinated cortical thickness measurements in a preliminary clinical study of 10 bipolar disorder type-I subjects and 10 healthy controls, and report significant decreases in the middle frontal gyrus in *T, G*, and *M* in the disorder, with the largest percentage change occurring in *M*. This study highlights the potential of myelinated cortical thickness measurements for investigating intracortical myelin involvement in brain disease at clinically relevant field strengths and resolutions.

## Introduction

The cerebral cortex contains a substantial number of myelinated axons whose pattern of distribution describes its *myeloarchitecture*. Intracortical myelin is found predominantly in the deeper layers of the cortex, and likely serves to speed the propagation of neural signals, as myelin does in the major white matter tracts. It also fine-tunes the timing and synchrony of neural networks, thereby continuously optimizing cortical function (Haroutunian et al., 2014). The density of these myelinated axons, their orientation, and how they are distributed over the cortical layers are regionally dependent in the cortex (Nieuwenhuys, 2013), suggesting that specific features of myeloarchitecture may be related to specific cortical functions. Thus, abnormalities in intracortical myelin may correlate with disrupted function in brain disease.

Magnetic resonance imaging (MRI) is now used *in vivo* to visualize intracortical myelin (Glasser and van Essen, 2011), since myelin alters MR parameters within the gray matter similarly to how it alters MR parameters in white matter. There have been several MRI studies linking changes in intracortical myelin to changes in function. Imaging studies in large numbers of subjects have confirmed cerebral myelin maturation and aging-related degradation (Grydeland et al., 2013; Shafee et al., 2015) and have indicated that a higher degree of intracortical myelin is associated with greater performance stability on a cognitive task (Grydeland et al., 2013). It has also been shown that the degree of myelination in the left posterior cortex plays a role in error processing and cognitive control (Grydeland et al., 2015), while a study in blind individuals suggests that increased intracortical myelin may represent a mechanism for compensatory functional reorganization in the visual cortex (Voss et al., 2014). Conversely, MRI has identified deficits in cortical myelin in mental disease, with losses of intracortical myelin being observed in patients with schizophrenia (Bartzokis et al., 2009, 2012). Combined, these studies predict a future role for MRI in studies of how intracortical myelin is related to function in health and disease.

While MRI lacks the resolution to visualize the fine details of the myeloarchitecture in living humans, it can map intracortical myelin content grossly across the entire cortex. For intracortical myelin mapping studies, the cortex is imaged with either a quantitative MRI sequence that measures a parameter that is sensitive to myelin [T_1_ (or R_1_), T${}_{2}^{*}$, magnetization transfer ratio (MTR); (Cohen-Adad et al., 2012; Marques and Gruetter, 2013; Sereno et al., 2013; Mangeat et al., 2015)], or a qualitative sequence with the contrast optimized such that the image intensity strongly correlates with the presence of myelin (T_1_-weighted; Bock et al., 2013). We used T_1_-weighted contrast for this study, because we have previously shown with *in vivo* MRI and matched histology in non-human primates that T_1_-weighted signal increases are spatially correlated with myelin density in the cortex (Bock et al., 2009). An image consisting of a combination of weightings (T_1_ weighted/T_2_ weighted) may also be used (Glasser and van Essen, 2011). Since the highest density of myelinated axons occurs in the deeper layers of the cortex (Layers IV–VI), the parameter or signal intensity in the resulting image is interpolated and displayed on a surface computed at a specific depth in the cortex to represent best the intracortical myelin content over all regions. Changes in the parameter value or signal intensity are then interpreted as reflecting changes in myelin amounts.

In cortical mapping studies, data sampled at the middle depth of the cortex are often used to summarize cortical myelin; high resolution imaging studies, however, have shown that the appearance of a map of myelin content over the cortex is highly dependent on the cortical depth at which it is made (De Martino et al., 2014; Lutti et al., 2014; Tardif et al., 2015). This means that comparisons of myelin content using MR signal between healthy and disease populations should incorporate cortical depth in their analyses, which could be hampered by the need to find a common cortical depth in diseases where overall cortical thickness is altered.

**Figure 1:**
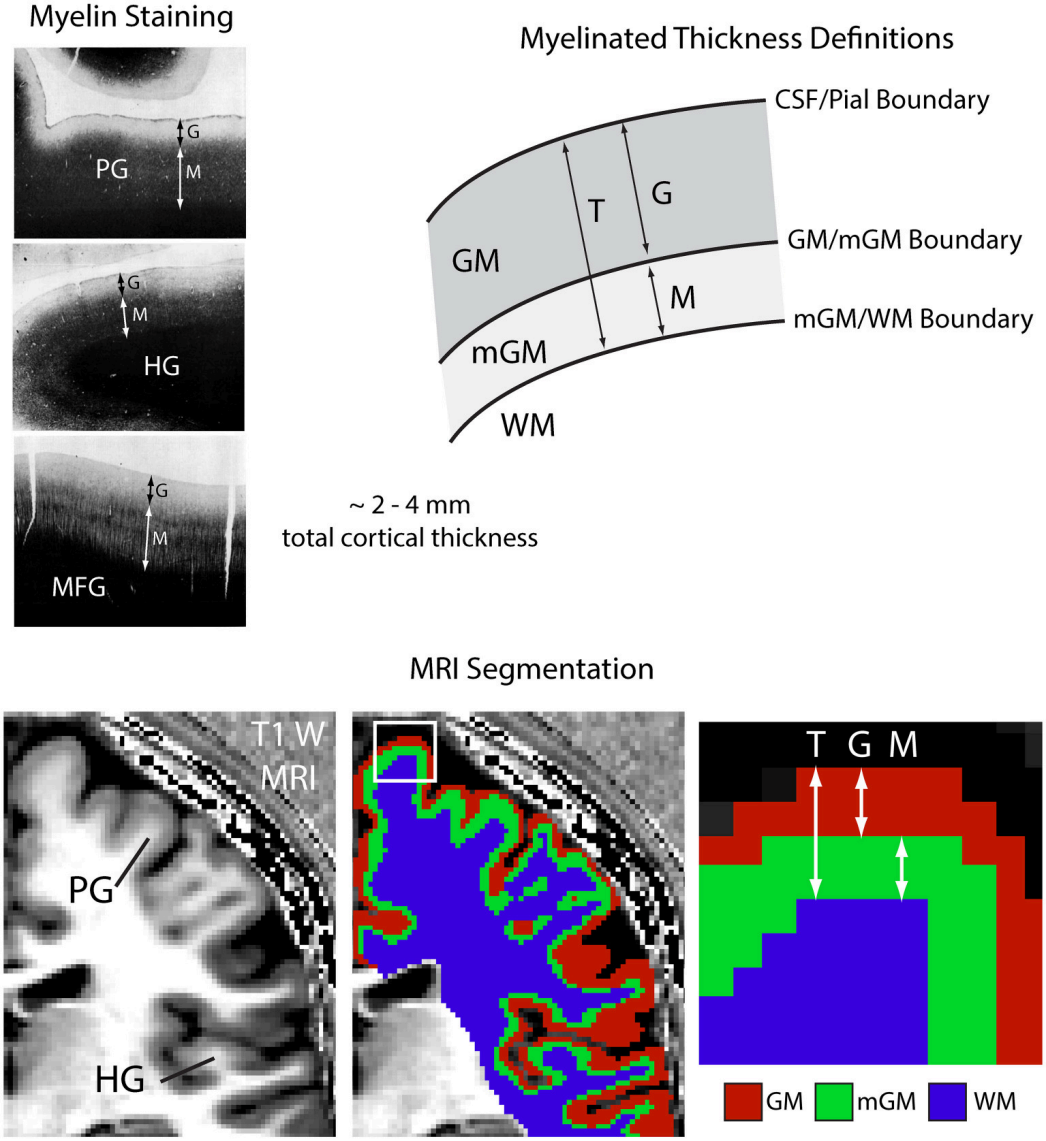
**Myelinated cortical thickness measurements**. The depth of the human cerebral cortex can be roughly subdivided into lightly myelinated and heavily myelinated portions, as seen in representative 40 μm thick histological sections stained for myelin (adapted from Braitenberg, 1962). This leads to intracortical contrast on heavily T_1_-weighted MRI, which can be used to segment the cerebrum into lightly myelinated gray matter (GM), heavily myelinated gray matter (mGM), and white matter (WM) tissue classes. Measuring the thickness of the GM and mGM tissue classes yields the thickness of the lightly myelinated (G) and heavily myelinated cortical layers (M). Overall cortical thickness (T) is also measured. The mGM class is thicker in regions known to be highly myelinated, such as the primary motor cortex in the precentral gyrus (PG) and the primary auditory cortex in Heschl's gyri (HG), MFG, middle frontal gyrus.

In this paper, we propose to investigate changes in cortical myelination based on morphology, rather than on MR signal changes. We do this by means of a myelinated cortical thickness technique, where we measure the thickness of lightly and heavily myelinated portions of the cortical depth separately. Separating cortical tissue into lightly and heavily myelinated portions based on MR contrast was previously suggested in 2D for studies of intracortical myelin in schizophrenia (Bartzokis et al., 2009), and here we extend it to map the entire cortex in 3D. Figure 1 shows representative 40 μm thick histological sections of human cortex stained for myelin (adapted from Braitenberg, 1962). In general, the deeper layers of the cortex (IV–VI) are typically the most heavily myelinated and will have an MR signal that is distinct from the signal in superficial gray matter, which has few myelinated fibers. There may be variations in these layers in some regions (where the Bands of Baillarger and the Stria of Gennari are not continuous with deeper heavily myelinated layers), but at the 1 mm isotropic resolution of clinical MRI, the cortical depth divides overall into a lightly myelinated and heavily myelinated portion. Thus, we propose to separate the traditional measure of total cortical thickness from neuroanatomical MRI into measures of the thickness of the lightly myelinated cortex (which we will refer to as “unmyelinated” cortex here for simplicity) and heavily myelinated cortex (referred to as “myelinated” cortex).

The myelinated cortical thickness measurement proceeds in three general steps:

1. Generate a weighted intensity image or a parametric map of the brain using MRI with strong intracortical contrast.
2. Identify the cerebrum (consisting of cortical gray matter and underlying major white matter tracts) in the brain and use a clustering algorithm to segment this anatomy into three tissue classes based on signal or parameter intensities: unmyelinated gray matter (GM), myelinated gray matter (mGM), and white matter (WM).
3. Use cortical thickness measurement techniques to find the distance between the cerebral spinal fluid (CSF)/GM boundary and the mGM/WM boundary (*T*), the distance between the CSF/GM and GM/mGM boundaries (*G*), and the distance between the GM/mGM boundary and the mGM/WM boundary (*M*).

These measurements yield three thickness parameters as shown in Figure 1: the total cortical thickness, *T*, the thickness of the unmyelinated layers, *G*, and the thickness of the myelinated layers, *M*. One may also calculate the proportional myelinated thickness of the cortex as:

(1) $$\begin{array}{l} P=\frac{M}{T} \end{array}$$

which summarizes the proportion of the cortical depth that is myelinated. The approach is similar to that used to measure total cortical thickness in standard software packages such as Freesurfer (http://surfer.nmr.mgh.harvard.edu/), except that it is considering three tissue classes in the cerebrum (GM, mGM, and WM), rather than two (GM and WM). This is only appropriate in the case that the input MRI image has enough intracortical contrast to allow segmentation into three classes.

In typical cortical thickness studies of brain disease, changes in total cortical thickness, *T*, are interpreted as broadly reflecting changes in cortical substructure caused by neurogenesis, axonal sprouting, dendritic branching, angiogenesis, and myelination (Zatorre et al., 2012). We expect that changes in the thickness of the myelinated portion, *M*, will be more specific to changes in intracortical myelin in brain disease than *T*, since that portion contains most of the myelin in the cortex.

Below, we demonstrate the feasibility of myelinated cortical thickness measurements made on T_1_-weighted images with strong intracortical contrast collected at a clinically-relevant field strength of 3 T. We show patterns of *T, G, M*, and *P* in representative atlases made from 20 healthy female subjects. We also demonstrate the utility of myelinated cortical thickness measurements in a preliminary study of 10 subjects with bipolar disorder and 10 healthy controls. The consensus in the literature is that bipolar disorder is associated with decreases in cortical thickness (Lyoo et al., 2006; Elvsashagen et al., 2013; Lan et al., 2014) and we question whether losses in intracortical myelin may underlie that thinning. That is in light of imaging (Marlinge et al., 2014; Lewandowski et al., 2015) and histology (Regenold et al., 2007) studies that indicate losses of myelin in major white matter tracts in bipolar disorder, and neuropathology studies indicating losses of the oligodendrocytes in cortical tissue that maintain myelin (Uranova et al., 2004; Savitz et al., 2014).

We performed two imaging studies: one in healthy controls to generate atlases showing normal myelinated cortical thickness patterns, and one in subjects with bipolar disorder type-I and healthy controls to see if we could detect differences in myelinated thickness in this mental disorder. Both studies used the same imaging procedure, image processing, image registration, and atlas generation techniques. We also performed histology in the cortex of a representative individual to examine myeloarchitecture in frontal regions relevant to bipolar disorder. For the bipolar study, we performed a region-of-interest analysis of T, G, and M in the middle frontal gyrus, a highly myelinated area that has been implicated in bipolar disorder in humans, and also in the precentral gyrus, an area containing motor regions thought to have little involvement in the disorder.

## Methods

### Imaging subjects

Studies were approved by the Hamilton Integrated Research Ethics Board and informed consent was obtained from each volunteer before enrollment. Images for the first study were collected in 20 healthy females, aged 30 ± 8 years (μ ± σ). Images for the second study were obtained in 10 healthy females, aged 30 ± 9 years, and 10 females with bipolar disorder type-I, aged 33 ± 8 years. Nine bipolar subjects had been clinically stable (euthymic) for at least 2 months and one was in a current depressive episode. All study participants were interviewed with the Structured Clinical Interview for DSM-IV (First et al., 2002) to confirm group status. In terms of treatment, three bipolar subjects were on lithium at the time of the study, four were on anticonvulsants, six were on atypical antipsychotics, two were on antidepressants and four were on anxiolytics. The average medication count across all 10 bipolar subjects was 2.50 (SD: 1.72), which is well-consistent with studies in bipolar disorder. The demographic details are presented in Table 1.

**Table 1:** **Subject demographics for the bipolar study presented as mean ± standard deviation**.

	**Control**	**Bipolar disorder type 1**
Number	10	10
Age	32.8 ± 9	33.3 ± 7
Education (years)	15.8 ± 2	16.2 ± 3
Height (inches)	64.7 ± 3	65.1 ± 3
Weight (pounds)	141 ± 34	157 ± 24
BMI (kg/m^2^)	23.7 ± 5	26.1 ± 3
Smoker	3	4
Duration of illness (years)	N/A	17.0 ± 7
Number of manic episodes	N/A	7.0 ± 5
Number of depressive episodes	N/A	15.2 ± 9
Number of mixed episodes	N/A	1.6 ± 3
Number of hypomanic episodes	N/A	9.3 ± 8
Age of first episode (years)	N/A	15.2 ± 6

### Imaging

Images were acquired on a 3 T General Electric scanner (Software Version 22.0) using a 32-channel receive-only coil for the head (MR Instruments) and a transmit body coil (GE).

### Anatomical reference image

A 3D T_1_-weighted whole-head image with 1 mm isotropic resolution was made using a 3D inversion-recovery gradient echo sequence (GE 3D BRAVO) [Inversion time = 450 ms, TE = 3.2 ms, TR in acquisition block = 8.4 ms, flip angle in acquisition block = 12°, field of view (FOV) = 25.6 × 25.6 × 25.6 cm, matrix = 256 × 256 × 256, linear phase encoding, Autocalibrating Reconstruction for Cartesian imaging (ARC) parallel imaging factor of 2 in the second phase encode direction, Number of averages = 1, time = 5 min 32 s]. This image served as an anatomical reference for image registration.

### High intracortical contrast T_1_-weighted image

Another 3D T_1_-weighted whole-head image with 1 mm isotropic resolution and increased intracortical contrast was made from four separate images collected with an inversion-recovery gradient echo sequence (GE 3D BRAVO) [Inversion time = 1100 ms, Time between end of acquisition block and next 180° pulse (TD) = 1000 ms, TE = 3.2 ms, TR in acquisition block = 8.4 ms, flip angle in acquisition block = 12°, FOV = 24.0 × 10.0 (selective slab in left/right direction) × 24.0 cm, matrix = 240 × 100 × 240, centric phase encoding, ARC factor 2 in second phase-encoding direction, Number of averages = 1, time = 5 min 53 s]. To increase intracortical contrast, each hemisphere was imaged separately. That reduced the matrix dimension in the first phase-encoding direction in the sequence, which in turn shortened the acquisition block following the inversion pulse. This is akin to using multiple segments in a magnetization-prepared rapidly-acquired gradient echo sequence (MP-RAGE; Mugler and Brookeman, 1990), where the shorter acquisition block improves contrast. A true MP-RAGE sequence was not available on our scanner, so we took this reduced-FOV approach instead, employing a long TD to increase intracortical contrast (Bock et al., 2013). Finally, to increase the contrast-to-noise ratio further, each hemisphere was imaged twice. Overall, this image took 24 min to acquire.

Each of the four separate images was registered to the anatomical reference image via a 6-parameter rigid transformation with sinc resampling using the FLIRT tool in FSL (Jenkinson et al., 2012) version 5.0 (fsl.fmrib.ox.ac.uk/fsl/). The magnitude images were then summed to create the final image of the whole head. This image for each individual was visually inspected for artifacts arising from improper registration, and none were found.

### Ratio image

A final 3D highly proton density-weighted whole-head image at 1 mm isotropic resolution was collected to correct B_1_ inhomogeneity artifacts in the T_1_-weighted image. The image was made with a 3D gradient-echo sequence (GE 3D SPGR) [parameters: TE = 3.1 ms, TR = 7.9 ms, flip angle = 4°, FOV = 24.0 × 17.4 × 24.0 cm, matrix = 240 × 174 × 240, Number of averages = 1, time = 5 min 29 s].

The proton-density weighted image was registered to the T_1_-weighted image using a 6-parameter rigid transform (FSL) and filtered with a 3D median filter with a 5 × 5 × 5 mm kernel size. The T_1_-weighted image was then divided by the filtered proton-density weighted image to create the ratio image, which is a highly T_1_-weighted image with B_1_– and some B_1_+ inhomogeneities removed (Wang et al., 2005; van de Moortele et al., 2009; Marques et al., 2010).

### Image processing

Image processing was performed predominantly in MIPAV v7.0.1 software (mipav.cit.nih.gov) using the JIST v3.0 (www.nitrc.org/projects/jist/), TOADS-CRUISE vR3c (www.nitrc.org/projects/toads-cruise/), and CBS High-Res Brain Processing Tools Version v3.0 (www.nitrc.org/projects/cbs-tools/) plug-ins, and Amira v5.2 software (Visage Imaging).

The first step in processing was to generate labels identifying the cerebrum in an individual's brain. The T_1_-weighted image was used as input to the SPECTER 2010 algorithm (Carass et al., 2011) in MIPAV, which created a mask identifying the brain and dura matter based on a combination of atlas-based segmentation, tissue classification and region growing. The T_1_-weighted image was used for this skull stripping rather than the Ratio image because the background in the Ratio image had a high mean value that made it difficult to identify air-tissue boundaries. The mask was then used to skull strip the Ratio image.

The skull-stripped Ratio image was used as input to the Multiple Object Geometric Deformable Model (MGDM) Multi-contrast Brain Segmentation algorithm (Bogovic et al., 2013; Bazin et al., 2014) in MIPAV to generate initial probabilistic labels for tissue classes in the brain. This algorithm evolves multiple boundary surfaces simultaneously according to competing forces derived from a prior intensity, shape and topology model to label the entire brain. The resulting probability labels for cerebral gray matter and white matter were then used as input to the CRUISE algorithm (Han et al., 2004) in MIPAV, which generated smoothed, topologically correct labels for the cerebrum using nested geometric deformable models and an anatomically consistent enhancement of sulcal CSF. This was performed in each hemisphere separately.

Subcortical structures and ventricles (as identified by the MGDM algorithm) were then removed from the labels for the left and right cerebrums and their labels were combined. This label for the entire cerebrum without the subcortical structures was then used to mask the Ratio image such that it contained only the cerebral cortex and underlying white matter tracts.

The cerebrum-masked Ratio image was then segmented into three tissue classes using the FANTASM algorithm (Pham, 2001) in MIPAV, which is based on a fuzzy c-means clustering algorithm with additional spatial regularization. This produced membership functions for each tissue class, whose voxel values described the probability of that voxel belonging to a particular class. For instance, a value of 0.5 or greater for a membership function would mean there was a greater than 50% probability of that voxel belonging to the given class. In order to define nested boundaries for the WM, mGM and GM, the membership functions were binarized with a series of thresholds: the first label, for the innermost mGM/WM boundary, was initially created to include all voxels with a given WM fuzzy membership threshold above 0.5. This was later refined to include all voxels with a given WM fuzzy membership threshold above 0.1 to reduce partial volume effects at the boundary with the WM. This refinement was performed to match more closely the WM segmentation that would result from a two-class tissue segmentation (see Section Results). Note that the fuzzy classification is independent of this adjustment, which only affects the location of the WM boundary. The mGM/GM boundary and unmyelinated thickness both remain unchanged. The second label, for the GM/mGM boundary, included the remaining voxels with an mGM fuzzy membership above 0.5, and finally the last label for the CSF/GM boundary included the remaining voxels again with a GM fuzzy membership above 0.5. All labels were split back into left and right hemispheres for subsequent processing. The mGM/WM-boundary labels were morphologically processed to remove all tissue not connected to the largest mass. The GM/mGM-boundary labels were slightly smoothed in Amira using 3D morphological smoothing with a kernel size of 2 × 2 × 2 mm. Voxels identified at the outermost surface of the cortex in these labels were removed using a mask made from a 1-voxel morphologically eroded version of the CSF/GM-boundary labels.

For distance measurements, signed distance levelset functions were calculated for the CSF/GM, GM/mGM and mGM/WM tissue boundaries using the Distance Field algorithm from the JIST plugins in MIPAV. Distances between boundaries were calculated as follows:

(2) $$\begin{array}{r} G=\varphi_{CSF∕GM}-\varphi_{GM∕mGM} \end{array}$$

(3) $$\begin{array}{r} T=\varphi_{CSF∕GM}-\varphi_{mGM∕WM} \end{array}$$

where φ is the levelset (the signed distance function perpendicular from a given surface) for a given boundary, *T* is the total cortical thickness, and *G* is the unmyelinated cortical thickness. These distances were measured at the CSF/GM (pial) surface and embedded in a 3D image of the same dimensions as the input images. This allowed sampling of the values at a surface generated at the pial surface.

The myelinated cortical thickness was calculated from these distance images as:

(4) $$\begin{array}{l} M=T-G \end{array}$$

such that *M* was also defined at the pial surface and propagated similarly.

Finally, the proportional myelinated thickness was calculated from the distance images according to Equation (1).

The distance images for individual subjects were visualized on the pial surface in Amira. This surface mesh was created from the CSF/GM boundary labels and the distance images were projected on it using nearest-neighbor interpolation.

### Average atlas generation

To create average atlases of *T, G, M*, and *P*, the distance images for each individual were registered to the ICBM 152 atlas space (Fonov et al., 2011) with FSL Version 5.0. The necessary warp was determined by non-linearly registering the T_1_-Weighted image to the ICBM 152 atlas using the parameters provided in the T1_2_MNI152_2 mm.cnf file, which is packaged with FSL. This warp was then applied to each distance file with sinc resampling to transform it to the atlas. The individual registered images were combined in Matlab (Mathworks) into a single average atlas image for each metric.

The registered atlas distance images were visualized in Amira using a surface mesh created from the pial surface of the MNI 152 atlas. This surface with the distance maps displayed on it was also inflated in Caret (van Essen et al., 2001; www.nitrc.org/projects/caret/) to show the pattern of myelinated thickness within the cortical folds. The cortical surface was inflated using 500 iterations with smoothing every 10 iterations and an inflation factor of 1.02.

### Histology

Postmortem brain tissue from a 43 year old male with a past history of depression and substance abuse was obtained from the Stanley Medical Research Institute's Brain Collection (Chevy Chase, MD). The tissue consisted of 10 μm thick paraffin embedded coronal sections of the dorsal lateral prefrontal cortex. For staining, sections were deparafinized and hydrated in 95% ethanol. They were then soaked overnight in Luxol® fast blue solution and differentiated until there was good gray/white matter contrast with light staining remaining in the gray matter. It was important not to differentiate overly the sections and remove all stain in the gray matter, since then the myelinated intracortical axons would not be visible. Whole stained sections were digitized using a Pentax K5 DSLR (Ricoh) with an SMC-A 50 mm f 2.8 macro lens. Captured color images were converted into grayscale in Adobe Photoshop CS4 using only the red channel, to enhance contrast. Magnified views of sections were captured on an EVOS® FL Cell Imaging System (Life Technologies) in gray scale using the Texas Red filter, again to enhance contrast.

### Region of interest analysis

For the bipolar study, average atlases of *T, G*, and *M* were created for control and bipolar groups. *T, G*, and *M* were then measured bilaterally in each individual's registered images in a region-of-interest (ROI) corresponding to the cortical ribbon in the middle frontal gyrus (MFG) and precentral gyrus (PCG). A statistical analysis was performed with the software Minitab 17 (Minitab, Inc.) separately on *T, G*, and *M* between control and bipolar groups in the MFG and PCG. A two-sided ANOVA tested for significant differences between groups at the *p* < 0.05 level, following confirmation that the data were normal (using an Anderson–Darling test) and that groups had equal variances. This was followed by a Tukey pairwise comparison test at a significance level of *p* < 0.05. A power analysis was also performed using the ROI data from the control subjects in Minitab to estimate effect size.

## Results

**Figure 2:**
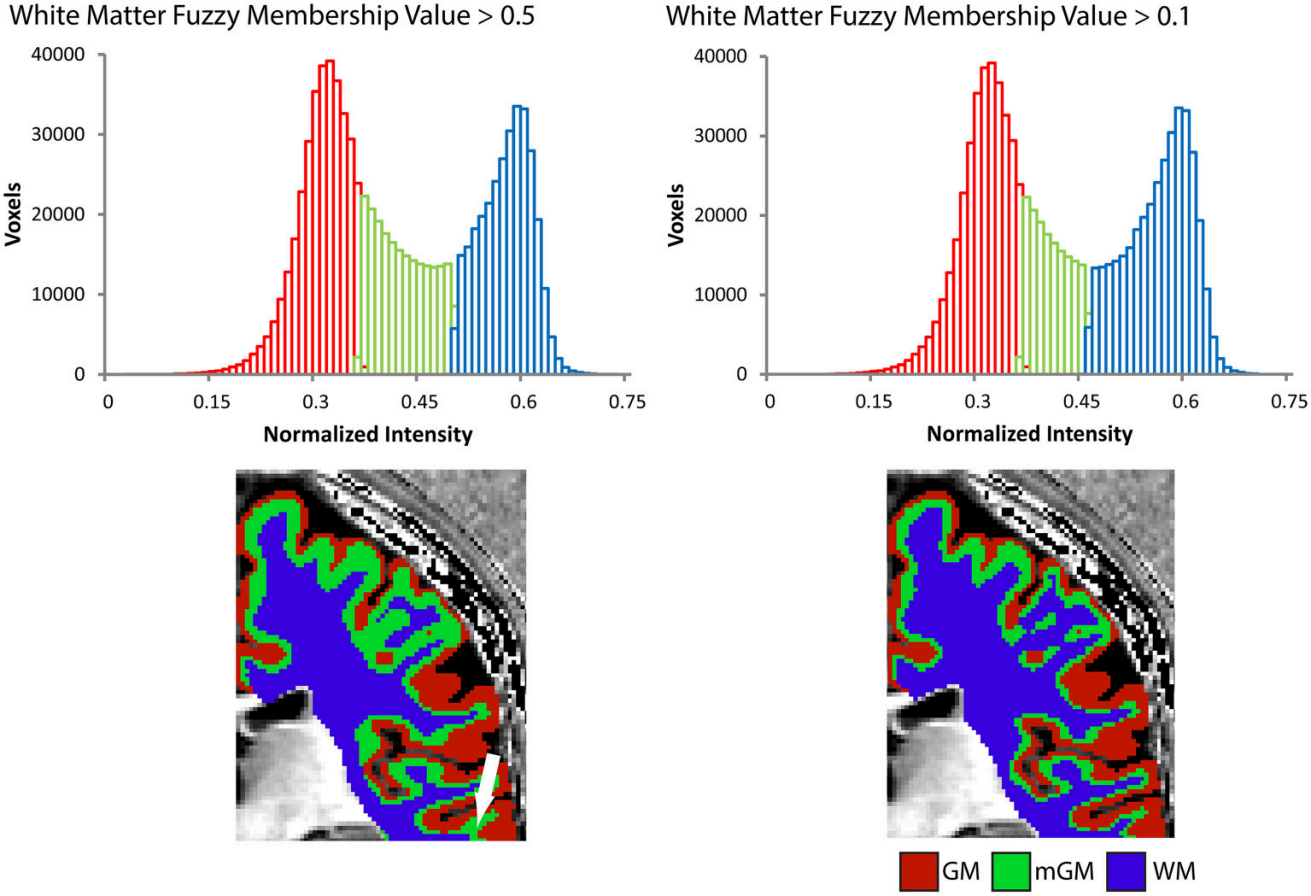
**Tissue segmentation results shown for a representative individual for WM fuzzy c-means threshold values greater than 0.5 or 0.1**. Each histogram plots the number of voxels assigned to each class in the cerebrum against normalized MRI signal intensity and each image shows the resulting segmentation superimposed on a 2D slice from the 3D Ratio MRI. Note the improved definition of the gGM/WM boundary in the thin gyral blade of white matter located at the white arrow.

The myelinated cortical thickness technique relies on data-driven clustering of MRI intensity in the voxels of the cerebrum to obtain the three tissue classes: GM, mGM, and WM. The FANTASM technique in our study is based on fuzzy c-means clustering, which means that each voxel can be assigned to multiple tissue classes. In our initial data analysis approach, we chose a fuzzy c-means membership threshold of greater than 0.5 for each tissue class, meaning that each contained voxels predominantly assigned to that class (Figure 2). We found, however, that this threshold underestimated the volume of the WM tissue class in the cerebrum because the additional mGM tissue class resulted in a displaced WM/mGM boundary compared to a two-tissue classification. This underestimation was especially prevalent in the thin gyral blades of white matter where partial voluming effects are strongest (see white arrow in Figure 2). Thus, we used a fuzzy c-means membership of greater than 0.1 for the WM class only, which we found to preserve better the geometry of the original WM boundary (see histograms in Figure 2). This had no effect on the GM/mGM boundary, as can be seen in Figure 2.

**Figure 3:**
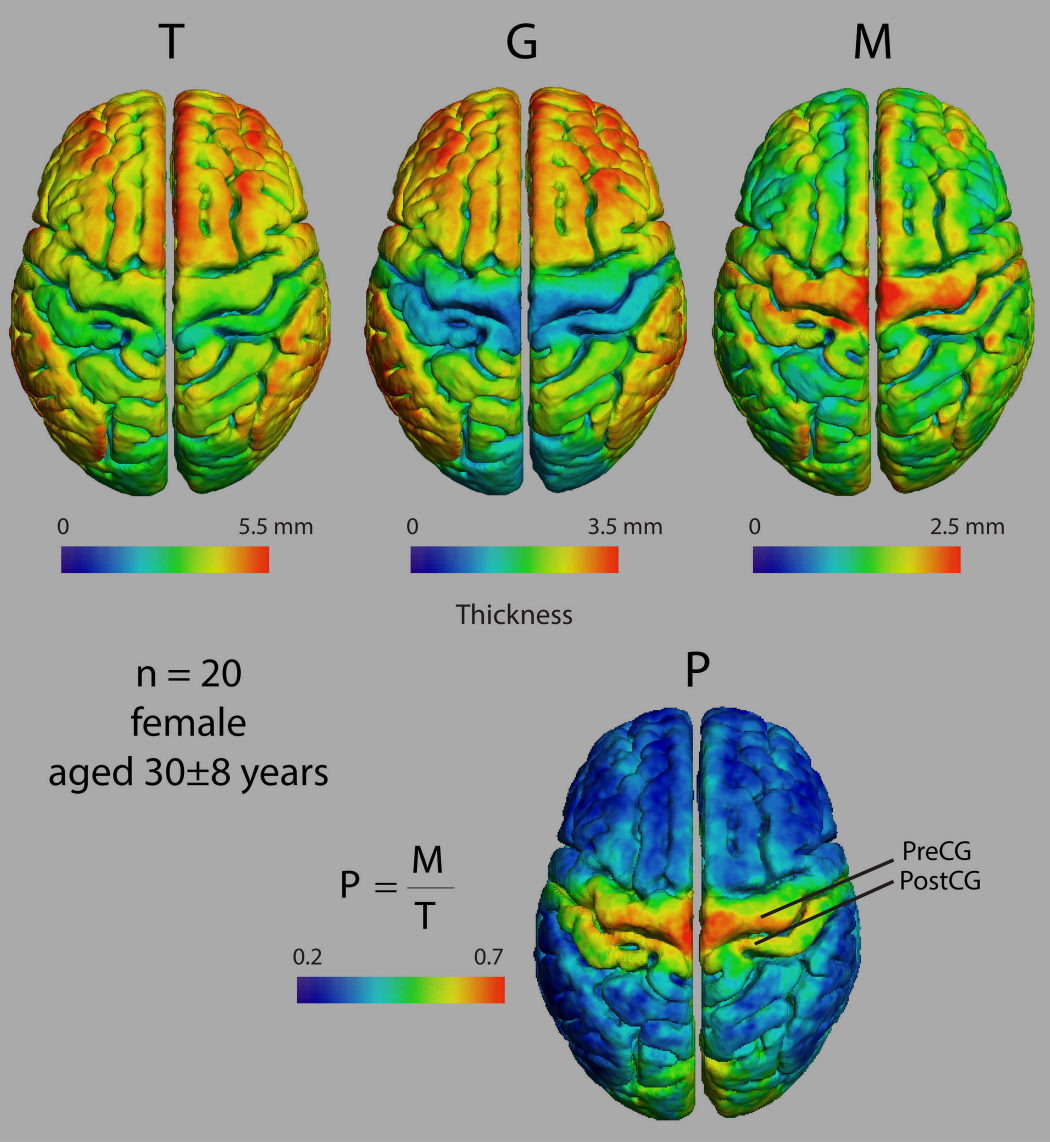
**Dorsal views of four thickness measures in an atlas made from 20 female subjects**. T, total cortical thickness; G, unmyelinated cortical thickness; M, myelinated cortical thickness; P, proportional myelinated thickness; PreCG, precentral gyrus; PostCG, postcentral gyrus.

**Figure 4:**
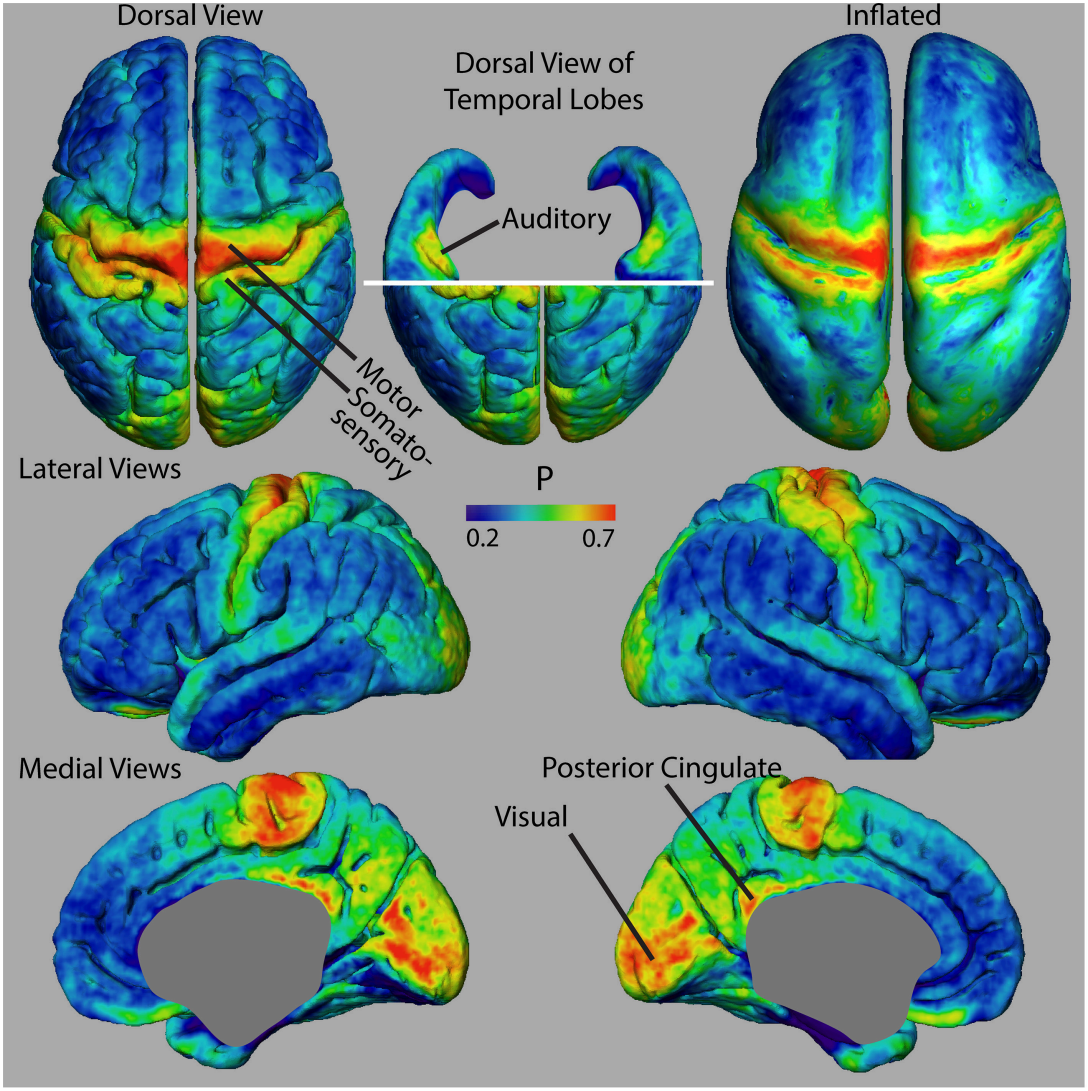
**Views of proportional myelinated thickness, P, in an atlas made from 20 female subjects**.

Figure 3 shows dorsal views of representative atlases of myelinated cortical thickness measurements in 20 healthy female subjects. It illustrates that the pattern of *G* over the cortex largely follows that of *T*, suggesting that cortical regions with the greatest total thickness also have the greatest thickness in their unmyelinated layers. The pattern of *M* does not follow *G* as closely and some regions that have the smallest total thickness, such as the postcentral gyrus and the calcarine fissure, in fact have the thickest myelinated layers. The figure also shows the atlas for *P*, which is a summary metric describing the proportion of the cortical thickness that is myelinated. It is interesting to note that regions showing the largest values for *M* and *P* are the same regions that have been identified as having high myelin contents from previous MRI studies of intracortical myelin using the MR signal for mapping (Glasser and van Essen, 2011; Cohen-Adad et al., 2012; Lutti et al., 2014). This is shown comprehensively in Figure 4 in views of the average atlas for *P* over the entire cortex, where the proportional depth of the cortex that is highly myelinated is greatest in the precentral gyrus (the location of M1 and associated motor areas), the postcentral gyrus (S1), Heschel's gyri (A1), around the calcarine fissure in the occipital cortex (the primary visual cortex, V1, and associated visual areas) and in the posterior cingulate gyrus. These regions are also described in the literature as having the highest myelin content as measured from histology (Hopf, 1956; Nieuwenhuys, 2013).

**Figure 5:**
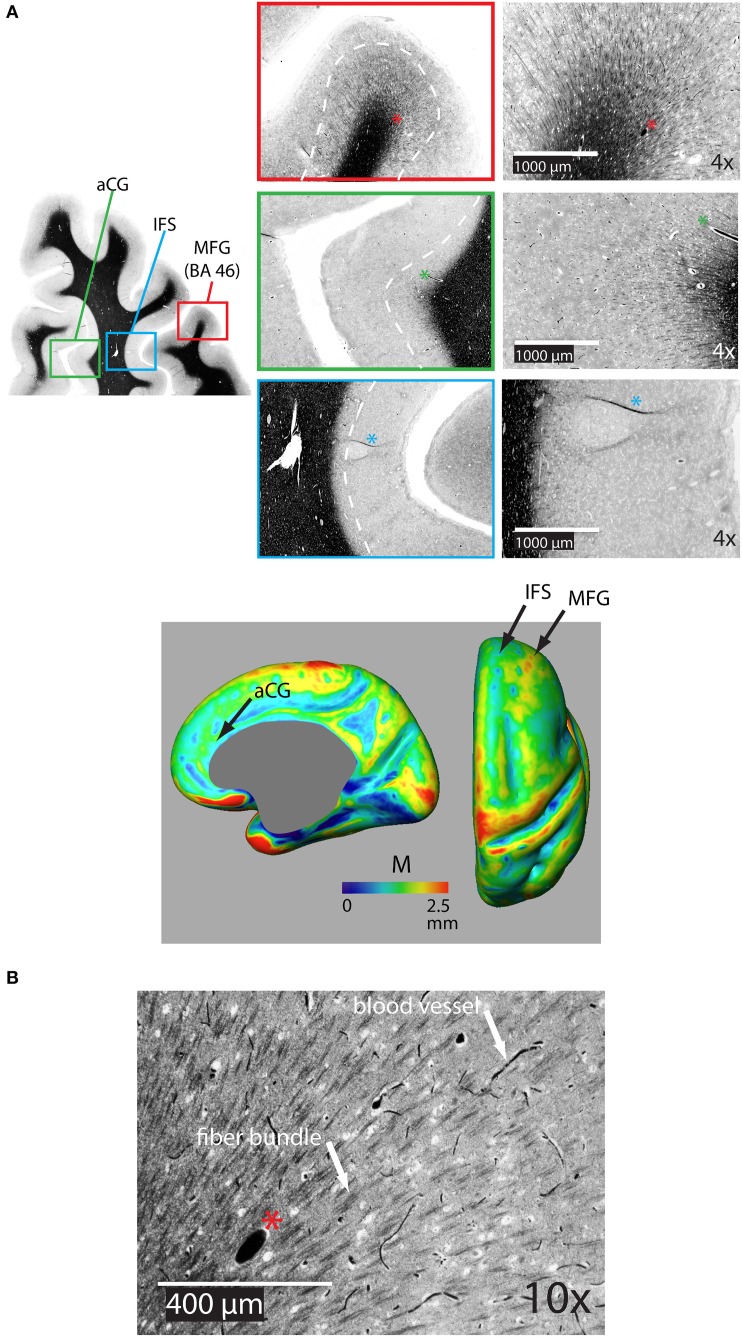
**(A)** Representative coronal section of the dorsal frontal cortex stained for myelin showing the distribution of bundles of myelinated fibers on the middle frontal gryus (MFG) [Brodman's area 46 (BA 46)], on the anterior cingulate gyrus (aCG), and in the inferior frontal sulcus (IFS). The white dotted line shows the rough extent of bundles over the three regions. The asterisk denotes the same blood vessel at each magnification. The bottom panel shows the location of regions on an inflated atlas of M in the right hemisphere in healthy controls on medial and dorsal views, respectively, pCG, posterior cingulate gyrus. **(B)** 10X magnification in the MFG illustrating bundles of myelinated fibers and distinctly darker blood vessels.

Figure 5A shows a representative distribution of myelinated fibers from histology in an individual in the dorsal frontal cortex. On the middle frontal gyrus (MFG), a dense distribution of myelinated fibers extends substantially through the cortical depth, implying that *M* would be thick in this region. That is confirmed in the *M* average atlas, which shows the MFG as having the greatest thickness of myelinated cortex in the dorsal frontal cortex (Figure 5A bottom). Figure 5B shows the myeloarchitecture in the MFG at a 10X magnification, revealing that most myelinated fibers exist in small bundles through the cortex. On the nearby anterior cingulate gyrus, the dense distribution of myelinated fibers does not extend as far over the cortical depth, implying that *M* is thinner in this region, which we also note in the average atlas. Finally, the dense distribution of myelinated fibers in the inferior frontal sulcus is closely localized at the WM boundary, suggesting that *M* is very thin in this region. Such is generally true of the depths of sulci in the cortex, which have been shown to contain far fewer myelinated fibers than the gyral crowns (Annese et al., 2004) and is again confirmed by low values for *M* in our average atlas.

**Figure 6:**
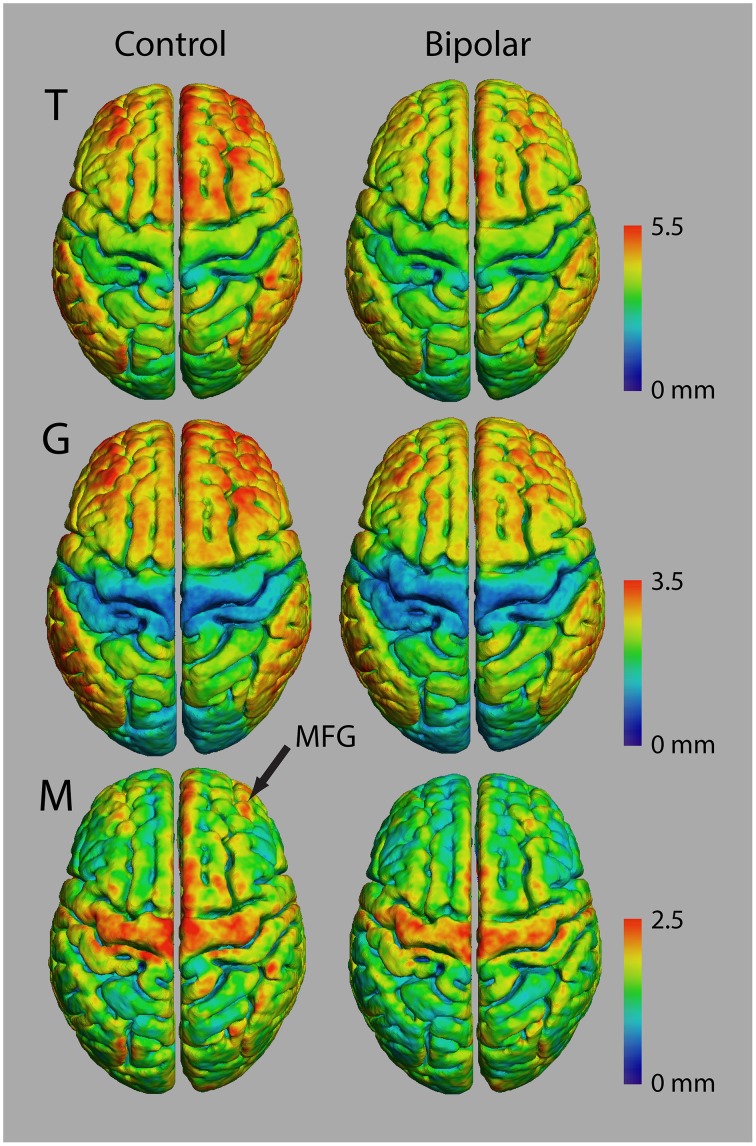
**Thickness measurements in controls and bipolar disorder type-I subjects (*n* = 10 in each group)**.

Figure 6 shows average atlases for *T, G*, and *M* in 10 control and 10 bipolar subjects from a preliminary study of myelinated cortical thickness in bipolar disorder. We did not include *P* in the analysis, because it includes both *M* and *T*, and thus is not an independent metric. The small sample size in this study does not produce adequate power for whole-brain statistical parametric mapping, but the trend in the maps of *T* suggests that total thickness is generally thinner in the bipolar subjects—more so in the association regions of the cortex than in the primary motor and sensory regions. The maps for *G* show largely the same trend, with visible decreases again generally in the association regions. The maps for *M* also show a trend toward thinner myelinated cortex in the bipolar subjects, although the decreases appear more regionally confined than for *T* and *G*.

**Figure 7:**
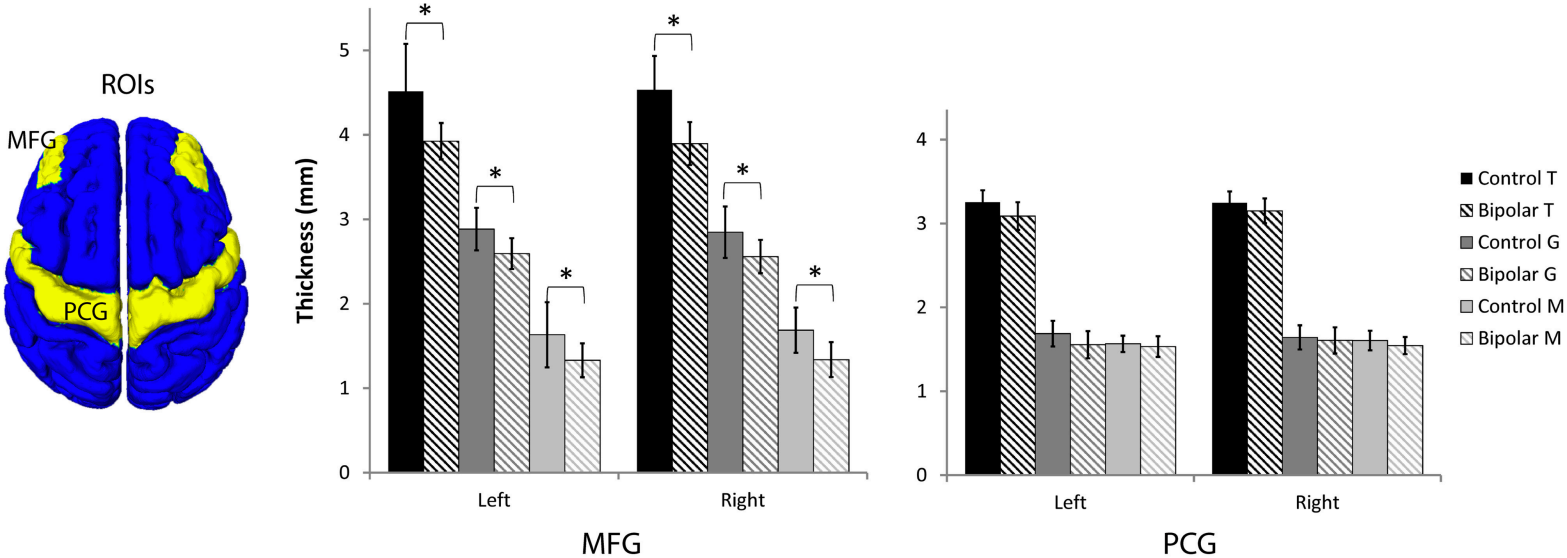
**Region-of-interest thickness measurements (μ ± σ, *n* = 10 in each group) in the middle frontal gyrus (MFG) and precentral gyrus (PCG)**. An asterisk denotes statistical significance between control and bipolar groups at *p* < 0.05.

Since the MFG in the dorsal lateral prefrontal cortex was shown to be highly myelinated in our normal average atlas of *M* and our histology study, we investigated changes there in a region of interest (ROI) analysis study of *T, G*, and *M* (Figure 7, Table 1, and Supplemental Figure 1). We also investigated changes in the precentral gyrus (PCG), a region that contains the primary motor cortex and is not thought to be involved in bipolar disorder. We found statistically significant decreases in all three metrics in bipolar subjects in both the left and right MFG (*p* < 0.05), but not in the PCG. Our observations of a reduced total cortical thickness in the MFG are consistent with other reports of cortical thinning in the DLPFC in bipolar disorder (Lyoo et al., 2006; Hartberg et al., 2011; Elvsashagen et al., 2013). Interestingly, the percentage difference change was largest for *M* of all the metrics, which could suggest that disruptions in intracortical myelin are implicated in the overall cortical thinning observed in other studies of the MFG in bipolar disorder.

Finally, we used the data from the ROI analysis for the 10 control subjects to estimate effect sizes for the myelinated cortical thickness technique. In the MFG, with a two-way *t*-test at a significance level of 0.05 and a power of 0.8, one could detect significant differences in mean *T* of 6%, *G* of 7%, and *M* of 11%. In the PCG, one could detect significant differences in the mean of *T* of 3%, of *G* of 6%, and of *M* of 5%. This suggests that the technique produces useful effect sizes for clinical studies, even in relatively small groups of individuals, provided the variance in thickness metrics in the clinical subjects was the same as the controls. We were actually able to detect smaller significant effect sizes in this study because the variance of the thickness values in the bipolar subjects was actually lower than in controls.

## Discussion and conclusion

Here we demonstrate that the cerebrum in an MRI image with high intracortical contrast can be segmented into three tissue classes using a clustering algorithm, which, based on brain histology, we hypothesize to represent lightly myelinated gray matter (GM), heavily myelinated gray matter (mGM), and white matter (WM). The mGM tissue class appears continuous across the deeper portion of the cortex at our imaging resolution of 1 mm isotropic resolution. Thus, with our technique we could define two unique thickness measures for shallower, unmyelinated gray matter and deeper, myelinated gray matter. This myelinated cortical thickness measurement provides a new approach to complement traditional cortical thickness studies where it is now possible to ascribe changes in cortical thickness in disease studies to one or both of the two tissue classes in the cerebral cortex.

Although 1 mm isotropic resolution may seem low for measuring meaningful changes in cortical thickness (since the cortex itself is only a few millimeters thick), it is still assumed to be possible to measure sub-millimeter changes in cortical thickness on 3D MRIs (Fischl and Dale, 2000). This is because the true underlying pial and white matter surfaces in the brain are smooth. Thus, if the radius of curvature of these surfaces and the thickness of the tissue classes is greater than the size of the imaging resolution, then the reconstructed surfaces can be interpolated to achieve sub-voxel accuracy in thickness measurements. The same argument holds true for myelinated thickness measurements, even though the unmyelinated and myelinated portions of the cortex are thinner than the overall cortex. This was validated by the power analysis in our study which showed similar effect sizes for measurements of *T, G*, and *M*.

The thickness of the myelinated layers, *M*, may be specific to changes in cortical myelination, and changes in this metric may indicate intracortical myelin involvement in brain diseases. For instance, *M* could change if either the number of myelinated fibers in the cortex, or the amount of myelin ensheathing their axons changed. Recent studies in mice have demonstrated the later, with socially isolated animals having thinner myelin sheaths around axons in the prefrontal cortex than control animals (Liu et al., 2012; Makinodan et al., 2012). Interestingly, the thinning of the myelin sheaths preceded changes in behavior, suggesting that changes in intracortical myelin represent plasticity in the cortex of isolated mice that leads to abnormal behavior. We confirmed reports of overall cortical thinning in the MFG in bipolar disorder, and further showed that the largest percentage change in thickness occurs in the myelinated depths of that region. The MFG area is located in the dorsal lateral prefrontal cortex, and is known to be associated with executive function. Since a number of meta-analyses have shown that individuals with bipolar disorder display poor performance in executive function even during periods of clinical remission of symptoms (Robinson et al., 2006; Bora et al., 2009; Bourne et al., 2013), these findings raise the possibility that disruptions in intracortical myelin may underlie in part the neurocognitive deficits seen in bipolar patients. With future studies in larger numbers of subjects, it will be possible to perform whole-brain analyses to see if other brain regions show similar thinning of *M* in the bipolar population. It will also be interesting to extend myelinated cortical thickness measurements to other brain diseases with noted changes in overall cortical thickness. Our preliminary findings of decreased intracortical myelin in bipolar disorder are in line with several studies using a variety of brain imaging techniques such as diffusion-tension imaging (Nortje et al., 2013), quantitative T_1_ρ mapping (Johnson et al., 2015), and magnetization transfer ratio (Lewandowski et al., 2015) showing abnormalities in the structure and myelin content of subcortical white matter in bipolar disorder. Because previous studies have found an association between white matter tracts and cognitive performance in bipolar disorder (Linke et al., 2013; Poletti et al., 2015), we speculate that changes in intracortical myelin in bipolar disorder may also reflect cortical plasticity that underlies some of the behavioral deficits and disorganized thought seen in bipolar disorder. A prospective study with repeated measures of intracortical myelin and clinical assessments over time would be necessary to test this hypothesis. While the underlying mechanisms of decreased intracortical myelin are yet to be determined, proposed mechanisms include increased oxidative stress, inflammation, loss of oligodendrocytes and blood-brain barrier dysfunction (Konradi et al., 2012; Andreazza et al., 2013; Patel and Frey, 2015).

The validity of the myelinated cortical thickness measurement technique depends on two main premises: (1) whether discrete boundaries between the proposed tissue classifications GM, mGM, and WM actually exist in the cortex and (2) whether one can image those boundaries accurately. Currently, there is no dataset in the literature quantifying the myelin content over the layers for the entire cortex to confirm the first point. A few studies exist, however, that measured the optical density of a myelin stain over the layers of the cortex in selected regions of the brain to quantify myelin content (Braitenberg, 1962; Hopf, 1968, 1969, 1970). These suggest that, for the examined regions of the brain, there is a division between unmyelinated and myelinated layers of the cortex that would approximately segregate into two discrete voxel intensities when imaged with MRI at least at a coarse resolution of 1 mm isotropic. We observed a similar division in our histology in the frontal cortex, where myelinated fibers were only seen to extend at a high density to a specific depth in each region. Whether this holds over the entire cortex is not clear, and there may be regions where the boundaries between unmyelinated and myelinated cortical layers are diffuse, making the proposed description of two cortical tissue classes too simplistic. The approximation of two classes of cortical tissue is worth exploring here, however, as a first step toward characterizing cortical myelination at clinically relevant resolutions in MRI. More elaborate models to describe cortical myelination are possible (Dinse et al., 2015), but currently these require sub-millimeter resolutions achievable only with ultra-high field MRI systems.

Even if discrete boundaries between tissue classes are present, the low image resolution in MRI leads to a mixing of tissue classifications, causing a partial voluming artifact in studies of cortical thickness. While partial voluming can cause any tissue boundary in thickness measurements to be incorrectly identified, the error is most pronounced at the mGM/WM boundary because of its high curvature and the thin geometric features of the WM volume. These effects can be lessened in sub-1 mm isotropic resolution images acquired with high field MRI (Lusebrink et al., 2013; Tardif et al., 2015). It would also be useful in future implementations of myelinated cortical thickness mapping to correct for partial volume effects using more sophisticated strategies than simply changing the WM fuzzy c-means membership function, as we did in this study (Pham and Bazin, 2004; Duche et al., 2014; Shafee et al., 2015).

There is also potential to refine further the myelinated cortical thickness technique and improve the quality of the thickness maps. For instance, although in this study we used an MRI protocol that was optimized to produce strong intracortical contrast, it was not specifically optimized for myelinated cortical thickness measures. Any segmentation routine based on clustering will perform more robustly the better the contrast-to-noise ratio (CNR) is between the three tissue classes; thus, different MR contrasts that are sensitive to myelin should be investigated to find the one that produces the best intracortical CNR.

Overall, measurements of myelinated cortical thickness provide a new description of intracortical myelination which can be measured at 3 Tesla in clinically achievable times. With the outlined methods, we were able to separate the cortex into lightly and heavily myelinated portions and this has the potential to disentangle changes in cortical myelination and overall cortical thickness in studies of aging, disease, and plasticity.

## Funding

This project was supported by a 2014 NARSAD Independent Investigator Grant from the Brain & Behavior Research Foundation (Dr. B. Frey), and an AFP Innovations Award, Department of Psychiatry and Behavioural Neurosciences, McMaster University (Dr. L. Minuzzi). This work was partially supported by the Marie Curie IRG grant 276684 (HIRESBRAIN7T).

### Conflict of interest statement

The authors declare that the research was conducted in the absence of any commercial or financial relationships that could be construed as a potential conflict of interest.

## Supplementary material

Supplemental Figure 1 **Individual region-of-interest thickness measures in the middle frontal gyrus (MFG) and precentral gyrus (PCG)**. The blue colored dot indicates the mean for each group.
