# Supplementary figures and images for: Assessing intracortical myelin in the living human brain using myelinated cortical thickness
Source: Front Neurosci. 2015 Oct 23;9:396. doi: 10.3389/fnins.2015.00396 (PMC4615825; doi:10.3389/fnins.2015.00396)

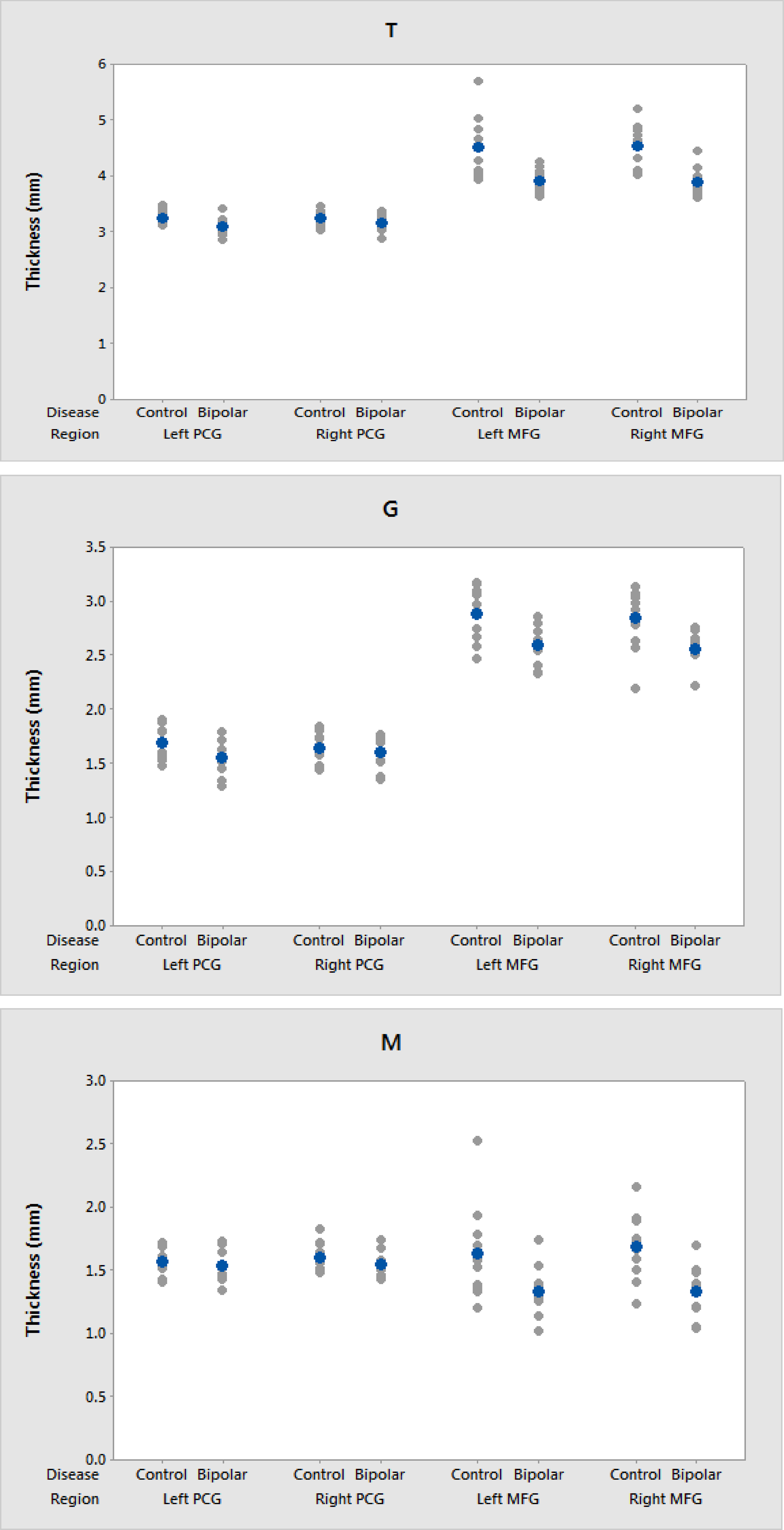

Supplement: Supplemental Figure 1 — Individual region-of-interest thickness measures in the middle frontal gyrus (MFG) and precentral gyrus (PCG). The blue colored dot indicates the mean for each group. [file Image1.TIF]
